# Supplementary material for: Association of Cardiometabolic Multimorbidity Pattern with Dietary Factors among Adults in South Korea
Source: Nutrients. 2020 Sep 7;12(9):2730. doi: 10.3390/nu12092730 (PMC7551044; doi:10.3390/nu12092730)
Supplement: Supplementary file 1 [file nutrients-12-02730-s001.pdf]

Table S1. Factor scores for multimorbidity patterns in adults aged 19 to 64 years in South Korea (KNHANES VI 2013-2015).

Disease	Factor 1 Cardiometabolic diseases pattern	Factor 2 Inflammatory disease pattern	Factor 3 Cancer and other diseases pattern
Obesity	**0.58**	−0.02	−0.15
Dyslipidemia	**0.64**	−0.01	0.02
Hypertension	**0.80**	−0.14	0.08
Allergic rhinitis	−0.11	**0.63**	−0.14
Diabetes mellitus	**0.64**	−0.10	0.03
Anemia	−0.04	−0.08	**0.29**
Osteoarthritis and osteoporosis	**0.45**	0.16	**0.40**
Depression	**0.26**	0.21	0.17
Atopic dermatitis	−0.16	**0.48**	−0.38
Pulmonary tuberculosis	0.16	−0.05	0.21
Thyroid disease	−0.02	0.07	**0.37**
Asthma	0.21	**0.51**	−0.08
Sinusitis	−0.05	**0.60**	0.21
Cancer [1]	0.01	−0.07	**0.57**
Otitis	−0.18	**0.50**	0.09
Stroke and CVD	**0.58**	0.00	0.10
Hepatitis B	0.04	0.07	**0.28**
Cataract	**0.36**	0.20	**0.46**

Statistical analysis was performed using exploratory factor analysis.[1] Cancer summed gastric, liver, colorectal, breast, uterine/cervical, lung, thyroid, and others.Bold indicates significance at factor score > 0.25. KNHANES: Korea National Health and Nutrition Examination Survey, CVD: cardiovascular disease.
